# Prohibition of Importation of American Cattle

**Published:** 1895-01

**Authors:** 


### EDITORIAL.

BELGIUM has just closed its ports against the importation of American cattle on the grounds of having discovered "contagious pleuro-pneumonia." This practically closes all the great centres of Europe, where American cattle have been in the past shipped in large numbers, and it is a grave reflection upon the Belgian veterinary authorities that they have fallen into the serious and grave error of charging the United States with having "contagious pleuro-pneumonia" within its borders. There are no veterinarians in the world more skilful and competent in the diagnosis of "contagious pleuro-pneumonia" than American veterinarians. They have had a training and experience second to those of no other country; and it is to be said to their honor and credit that they are the only veterinarians in the world who have successfully stamped out the disease wherever it has secured a foothold, and they challenge the world, and offer every facility that can be afforded to any committee or body of veterinarians representing the different countries of Europe to come here and find within our borders a single herd afflicted with "contagious pleuro-pneumonia." It is now more than three years since a single acute outbreak has been found, and more than two years since a single chronic case has been discovered. Our nation has spent thousands and thousands of dollars endeavoring to find some locality where the disease still prevailed; and has finally been compelled to reduce the force of veterinarians in the Bureau of Animal Industry to an insignificant number, because of there being nothing for them to do in this direction, which was the chief line of their employment for a number of years. It is a very serious and grave reflection upon the veterinary profession abroad, and must in the end work incalculable harm to those countries upon the recommendation of whose veterinarians exclusion of American cattle has been advised, and will, in the future, greatly lessen them in the estimation of the younger members of the profession here whose ambition it has been to spend a portion of their college life in some one of the foreign schools. It will be but natural that their confidence must be weakened when they are confronted with the fact that with all their boasted advancement in veterinary science they have not yet learned to diagnose accurately "contagious pleuro-pneumonia," which has been a bovine scourge of many countries for scores of years.

